# Unraveling molecular characteristics and tumor microenvironment dynamics of neuroendocrine prostate cancer

**DOI:** 10.1007/s00432-024-05983-0

**Published:** 2024-10-16

**Authors:** David Heimdörfer, Nastasiia Artamonova, Zoran Culig, Isabel Heidegger

**Affiliations:** Department of Urology, Medical University Innsbruck, Innsbruck, Anichstreet 35, Innsbruck, A-6020 Austria

**Keywords:** Neuroendocrine prostate cancer, Tumor microenvironment, Molecular alterations, Personalized treatment

## Abstract

Prostate cancer (PCa) is the most prevalent malignancy and the second leading cause of cancer-related deaths among men. While adenocarcinoma of the prostate (adeno-PCa) is well-characterized, neuroendocrine prostate cancer (NEPC) remains poorly understood. Generally, NEPC is a rare but highly aggressive histological variant, however its limited patho-physiological understanding leads to insufficient treatment options associated with low survival rates for NEPC patients. Current treatments for NEPC, including platinum-based therapies, offer some efficacy, but there is a significant need for more targeted approaches. This review summarizes the molecular characteristics of NEPC in contrast to adeno-PCa, providing a comprehensive comparison. A significant portion of the discussion is dedicated to the tumor microenvironment (TME), which has recently been identified as a key factor in tumor progression. The TME includes various cells, signaling molecules, and the extracellular matrix surrounding the tumor, all of which play critical roles in cancer development and response to treatment. Understanding the TME’s influence on NEPC could uncover new avenues for innovative treatment strategies, potentially improving outcomes for patients with this challenging variant of PCa.

## Literature review

We reviewed literature until 15. September 2024 (PubMed, Google Scholar) and conference reports from major urological and oncological meetings from the last five years (annual meeting of the American Society of Clinical Oncology (ASCO), ASCO Genitourinary Cancers (ASCO GU), European Association of Urology, American Urological Association (AUA), American, Association for Cancer Research (AACR)) up until the ESMO meeting 2024. The inclusion criteria encompassed studies in English including at least one of the following key words for initial search: “neuroendocrine prostate cancer”, “primary neuroendocrine prostate cancer”, “de novo neuroendocrine cancer”, “de novo primary prostate cancer”, “tumor microenvironment”, “treatment”, “single cell RNA sequencing”, “scRNA-seq” and “spatial transcriptomics”.

Among others, optional keywords such as “cancer associated fibroblasts”, or “check point inhibitors” have been later included for a more detailed description of the respective pathways or fields of research.

## Current treatment strategies for NEPC

Prostate cancer (PCa) is the most prevalent malignancy among men and the second leading cause of cancer-related mortality in this population (Siegel et al. 2020). While over 95% of prostate tumors are characterized as adenocarcinomas, histological types such as sarcomatoid variants or neuroendocrine prostate cancers (NEPC) are rare forms of the disease (Lavery et al. 2016).

In recent years, there has been growing awareness in the field of PCa research that adenocarcinomas can evolve into NEPC after androgen deprivation therapy (ADT) by luteinizing hormone-releasing hormone (LHRH) treatment, (Le et al. 2023), which is one of the most common treatment options for PCa (Choi et al. 2022). Additionally, androgen receptor pathway inhibitors (ARPIs), such as abiraterone and enzalutamide, have been developed as alternative therapeutic options (Choi et al. 2022; Ong et al. 2023). The transformation to NEPC can occur either due to alterations in androgen receptor (AR) signaling or the acquisition of a neuroendocrine phenotype, leading to a condition known as treatment-emergent neuroendocrine prostate cancer (T-NEPC) (Beltran et al. 2019a), which is observed in approximately 17–30% of castration-resistant prostate cancer (CRPC) cases (Le et al. 2023). This transformation is particularly problematic as the tumor no longer relies on androgen signaling, rendering conventional treatments like ADT and ARPI ineffective (Wang et al. 2021b).

In contrast, *de novo* NEPC is a very rare but highly aggressive subtype of PCa that comprises about 2% of all PCa cases (Liu et al. 2022). Importantly, when NEPC arises *de novo*, it is mostly diagnosed at an advanced metastatic stage (Zhu et al. 2021), contributing to its aggressive clinical behavior and resulting in a median overall survival rate of only 16.8 months (Conteduca et al. 2019). Current treatment options for NEPC are primarily limited to platinum-based chemotherapies, such as cisplatin or carboplatin (often combined with etoposide) (Vlachostergios and Papandreou 2015; Artamonova et al. 2024).

Besides the limited efficacy of platinum-based therapies, treatment can be accompanied by significant side effects including hematological complications, fatigue, nephrotoxicity or cardio-vascular events (Gupta et al. 2016; Corn et al. 2019; Duan et al. 2020), highlighting the urgent need for novel therapeutic strategies for NEPC patients. Another problem is the reduction of renal function with rising age (measured by glomerular filtration rate (GFR), which favors nephrotoxicity of renal excreted drugs such as cisplatin or carboplatin (Lichtman et al. 2007). Furthermore, a general reduction of the dose is recommended for these drugs with GFR levels below 60 ml/min, whereby the application of cisplatin is not recommended in these patients (Lichtman et al. 2007). The lack of a standard second-line treatment following platinum-based therapy for *de novo* and T-NEPC patients results in a poor prognosis (Eule et al. 2023) and poses clinical challenges in the daily practice.

Aggressiveness of PCa with higher rates of metastasizing and poor outcome have been linked to germline mutations of *BRCA1* and *BRCA2 (BRCA1/2)* (Castro et al. 2013). Furthermore, *BRCA2* alterations have been shown to be relatively common in PCa with a NEPC phenotype, since homo- and heterozygote mutations or deletions of *BRCA2* comprised about 29% in CRPC with a NEPC phenotype in one study (Beltran et al. 2019a), while in another patient cohort biallelic *BRCA2* alterations appeared in 26 and 9% in NEPC and non-NEPC PCa patients, respectively (Symonds et al. 2022). Inhibition of poly(ADP-ribose) polymerases (PARPs), which play a critical role in DNA repair, has emerged as a promising therapeutic strategy for tumors harboring *BRCA* mutations (Curtin and Szabo 2020). PARP inhibitors (PARPi) have demonstrated efficacy as monotherapy or in combination with novel hormonal therapies (NHT) in mCRPC (Teyssonneau et al. 2024). However, the case studies of NEPC patients with *BRCA1/2* mutations who underwent PARPi maintenance therapy with olaparib showed variable outcomes (Turina et al. 2019; Pandya et al. 2021; Kaitsumaru et al. 2023). Besides to the favorable tumor responses reported by Turina et al. (2019) and Kaitsumaru et al. (2023), also adverse events such as interstitial pneumonia (Kaitsumaru et al. 2023) or limited efficacy, likely due to *BRCA2* reversion mutations, have been reported (Pandya et al. 2021). Therefore, the efficacy of PARPi in treating NEPC with *BRCA1/2* mutations requires further investigation and evaluation. Additionally, the treatment of NEPC by a combination of the PARPi olaparib and cyclin-dependent kinase inhibitors targeting CDK4/6 (Wu et al. 2021) or CDK2/5 (Liu et al. 2019) was already suggested as a promising line of therapy for NEPC. In both studies a decrease of neuroendocrine gene expression was observed (Liu et al. 2019; Wu et al. 2021), while the application of olaparib and palbociclib or abemaciclib also reduced tumor growth and induced apoptosis (Wu et al. 2021).

Recent advancements in cancer treatment have highlighted the potential of immunotherapy, particularly checkpoint inhibitors, which have shown efficacy in treating small cell lung cancer and other extrapulmonary neuroendocrine tumors (Stelwagen et al. 2021; Pokhrel et al. 2022). However, as known from trials of advanced adenocarcinomas of the prostate, including NEPC differentiation (Pokhrel et al. 2022), only a certain subset of patients benefits from immunotherapy (Heidegger et al. 2021; Pokhrel et al. 2022). Other studies suggest that NEPC exhibits high levels of molecular and genetic heterogeneity, with certain molecular signatures potentially conferring sensitivity to PD-L1 inhibition (Yoshida et al. 2022; Wen et al. 2023). Moreover, T-cell depletion appears more pronounced in NEPC compared to prostate adenocarcinoma, with only a minority of NEPC tumors being inflamed (Bhinder et al. 2023).

Another suggested therapy target is delta-like ligand 3 (DLL3), which was shown to be overexpressed in NEPC (Puca et al. 2019). Tartalamab, a bispecific T-cell engager that binds DLL3 and CD4, is currently tested in a phase 1b study in patients with *de novo* or treatment-emergent NEPC (Aggarwal et al. 2024). Despite its innovative approach, only 10.5% of the overall patient population responded to the treatment, although higher response rates were observed in DLL3-positive tumors (Aggarwal et al. 2024). Altogether, these findings underscore the necessity for a deeper understanding of the molecular landscape of NEPC, which could lead to the development of more personalized and effective therapeutic strategies.

### Histopathological differences among adeno-PCa and NEPC

#### Adeno-PCa

As mentioned above, the most common histological subtype of PCa is the adenocarcinoma which mostly emerges within the peripheral zone of the prostate (Lavery et al. 2016). Characterized by a luminal phenotype with a lack of basal cells and strong AR signaling, adenocarcinomas were long thought to originate from luminal cells (Park et al. 2016). However, it has been previously shown that basal cells can divide symmetrically into two basal or asymmetrically into a basal and a luminal daughter cell; whereas cell division of luminal cells is only symmetrical (Wang et al. 2014). In line with this, recent studies revealed that adenocarcinomas may originate from either basal (Goldstein et al. 2010; Stoyanova et al. 2013; Park et al. 2016) or luminal cells, potentially leading to distinct PCa subtypes (Park et al. 2016). The luminal phenotype predominates adenocarcinomas of the prostate, which are mostly characterized by a loss of basal cells and high AR signaling (Park et al. 2016). While prostate-specific antigen (PSA) is regulated by AR and often exhibits elevated levels in PCa, it is rather prostate tissue-specific than cancer-specific (Kim and Coetzee 2004). Yet, it has remained the most valuable biomarker for PCa detection and surveillance for many years (Kim and Coetzee 2004).

#### *De novo* NEPC

Neuroendocrine cells are terminally differentiated and comprise about 1% of the prostate epithelium, with lower abundance in the central zone compared to the transitional and peripheral prostate zones (Butler and Huang 2021). While Aumüller et al. hypothesized that neuroendocrine cells migrate into the prostate epithelium from the neural crest during embryogenesis (Aumüller et al. 1999), other studies suggest that neuroendocrine cells might rather differentiate from basal cells due to lineage plasticity (Bonkhoff et al. 1994; Rumpold et al. 2002).

Neuroendocrine cells exhibit dendrite-like structures that can extent between the stroma and the epithelial layer (Abrahamsson 1996). These cells can grow between other epithelial cells and appear as open or closed morphological subtypes, which do or do not reach into the lumen, respectively (Abrahamsson 1996). In the prostate epithelium, neuroendocrine cells can be well discriminated from their surrounding cells by IHC (Butler and Huang 2021), especially by chromogranin A (CHGA), synaptophysin (SYP), or CD56 (NCAM) staining (Epstein et al. 2014). While neuroendocrine cells do not express AR, PSA and Ki-67, they secrete several cytokines, hormones and growth factors, such as neural growth factor (NGF), histamine, vasoactive intestinal peptide, parathyroid hormone-related protein, vascular endothelial growth factor, calcitonin, neuropeptide Y, bombesin/gastrin-releasing peptide, serotonin, somatostatin, Interleukin 8 (reviewed by Butler and Huang 2021). Notably, luminal cells express the respective receptors for the latter six of these factors (reviewed by Butler and Huang 2021). In addition, neuroendocrine cells are often located in proximity of proliferating Ki-67 positive epithelial cells, which can be explained by their secretion of several factors contributing to cell growth and angiogenesis (Arman and Nelson 2022). Hence, they have been suggested to be important mediators not only of prostate growth and differentiation, but also of the epithelial secretory function (Fine 2018).

According to the Prostate Cancer Foundation in 2013, several subtypes of PCa with neuroendocrine differentiation can be distinguished, such as prostate adenocarcinoma with neuroendocrine differentiation, adenocarcinoma with Paneth cell neuroendocrine differentiation, carcinoid tumor, small cell carcinoma (SCPC), large cell neuroendocrine carcinoma (LCNPC), and mixed (small or large cell) neuroendocrine carcinoma as well as acinar adenocarcinoma (Epstein et al. 2014). With a focus on pure neuroendocrine phenotypes of the tumors, of which SCPC is the most frequently-appearing, carcinoid tumors are PSA negative, express a diffuse pattern of neuroendocrine markers (SYP, CHGA, NCAM), and have only a 5–20% or less increased Ki-67 proliferation rate (Epstein et al. 2014). While over 90% of SCPC appear positive for at least one of the neuroendocrine markers, those are only diffusely expressed in LCNPC cells (Epstein et al. 2014). However, both SCPC an LCNPC are very aggressive, mostly negative (or sparsely focally positive) for PSA, and highly proliferative (Epstein et al. 2014). In addition, SCPC cells have been shown to express TTF-1, CD56, Bcl-2, and c-Kit, with a mainly neuroendocrine phenotype (Yao et al. 2006).

#### Development of T-NEPC

Over 90% of diagnosed prostate cancers are adenocarcinomas with a luminal phenotype responsive to androgen deprivation therapy (ADT) inhibiting the highly AR signaling dependent tumor growth (Wang et al. 2021b). However, PCa can develop ADT resistance either by alterations of AR signaling, including AR gene amplification, genomic mutations or rearrangements, or by a phenotypical shift to AR-signaling independent T-NEPC (Beltran et al. 2019a). Hence, the development of T-NEPC has been a highly discussed field in PCa research for a few years. Recently, Merkens et al. (2022) summarized the molecular mechanisms of the development of T-NEPC, which has been experimentally determined to potentially arise from different cellular origins. The most common way is probably the transdifferentiation of luminal adeno-PCa cells into neuroendocrine cells, while also clonal evolution from basal or neuroendocrine cells was described. Furthermore, T-NEPC transdifferentiation is considered a highly complex process, where besides differential expression of neuroendocrine markers, also predominant deregulations in markers for lineage-plasticity, proliferation, epithelial-to-mesenchymal transition, and angiogenesis have been described (Merkens et al. 2022). Key events in molecular alterations during neuroendocrine transdifferentiation include but are not limited to *RB1* loss, *PTEN* loss, *TP53* loss, *MYCN* amplification, upregulation of several transcription factors (e.g., *SOX2*, *ONECUT2* and *PEG10*), epigenetic regulators (e.g., EZH2 and Heterochromatin protein 1α), or downregulation of the transcription factors RE1 Silencing Transcription Factor or FOXA1 (Wang et al. 2021b). Many of these molecular changes ultimately manifest in molecular hallmarks of NEPC, some of which are being discussed in the next paragraph.

#### Molecular hallmarks of NEPC

Of importance, genetic alterations were only rarely specifically described in *de novo* NEPC claiming for basic research trials investigating the molecular landscape of *de novo* NEPC. The primary objectives were to enhance the understanding and characterization of de novo NEPC and to discuss new therapeutic targets since current therapeutic implications are very limited.

Regarding aggressive T-NEPC, the transcription factor ONECUT2 has been determined as a transcriptional regulator of neuroendocrine transdifferentiation (Guo et al. 2019). Furthermore, ONECUT2 overexpression was demonstrated to positively correlate with hypoxia and promote neuroendocrine differentiation in LNCaP cells by activating *ASCL1*, *PEG10*, and *NSE* expression (Guo et al. 2019). Importantly, two independent studies determined two T-NEPC subtypes based on their differential expressions of either ASCL1 and NEUROD1 (Cejas et al. 2021) or ASCL1 and CHGB (Wang et al. 2022) as marker genes of the respective subgroup. Furthermore, Chen et al. (2023a) could demonstrate genetic heterogeneity of NEPC in an AR-independent in vivo temporal transformation model. They observed two classes of NEPC, which were characterized by ASCL1 and ASCL2 and POU2F3 expression (Chen et al. 2023a). Cejas et al. (2021) pointed out that the inter- and intra-tumoral heterogeneity of NEPC phenotypes gives rise for adapting therapy strategies in a case dependent manner.

Gene fusions involving *TMPRSS2* and *ERG* or *ETV1* frequently occur in PCa, leading to androgen-dependent over-activation of oncogenic ETS family member proteins (Tomlins et al. 2005). Notably, *ERG* rearrangements are found in 45–86% of SCPC cases, but they do not occur in small cell lung cancer (Scheble et al. 2010; Guo et al. 2011; Lotan et al. 2011) or small cell bladder cancer (Guo et al. 2011), highlighting their potential uniqueness in PCa. Although fluorescence in situ hybridization used in these studies did not directly confirm ERG fusion with TMPRSS2, Guo et al. identified TMPRSS2-ERG fusion genes in two xenografts with ERG rearrangement via deletion (Guo et al. 2011). Future studies should examine whether *TMPRSS2* and *ERG* alterations are also prevalent in *de novo* NEPCs.

While overexpression of the serine/threonine Aurora A kinase (AURKA) was correlated with centrosome amplification and chromosomal instability in multiple cancer types (Zhou et al. 1998), in neuroblastoma Aurora A was shown to directly bind to N-myc and thereby protecting it from ubiquitin-dependent proteasomal degradation (Otto et al. 2009). Beltran and colleagues observed a higher expression of *AURKA* and *MYCN* in NEPC than in adeno-PCa (Beltran et al. 2011). Importantly, their data suggest that both genes are implicated in neuroendocrine differentiation, since knockdown of *AURKA* resulted in reduced expression of *NSE* in the NCI-H660 NEPC cell line, and stable overexpression of *MYCN* in LNCaP cells induced a phenotype with neuroendocrine features, possibly due to its binding to the promoters of *NSE*, *SYP*, and *AR* (Beltran et al. 2011). It was later shown that overexpression of N-MYC and myristoylated AKT (myrAKT), a Serine/Threonine kinase involved in the inhibition of apoptosis and cell proliferation (Wu et al. 2014), in human prostate basal cells resulted in adenocarcinoma and NEPC (Lee et al. 2016). Interestingly, both PCa types showed a gene expression profile similar to earlier described NEPC (Beltran et al. 2011; Lee et al. 2016). In addition, ADT induces overexpression of the transcription factor ZBTB46 (Chen et al. 2017), which causes a muscarinic acetylcholine receptor 4 (CHRM3) dependent activation of MYCN/AKT and differentiation to T-NEPC through enhanced expression of nerve growth factor (NGF) (Chen et al. 2021). Furthermore, high *NGF* and *CHRM3* expression correlated with higher Gleason scores in general, whereby the highest expression levels were observed in SCPC (Chen et al. 2021). These data give rise to the question about a potential tumorigenic role of ZBTB46 in *de novo* NEPC.

**Fig. 1:**
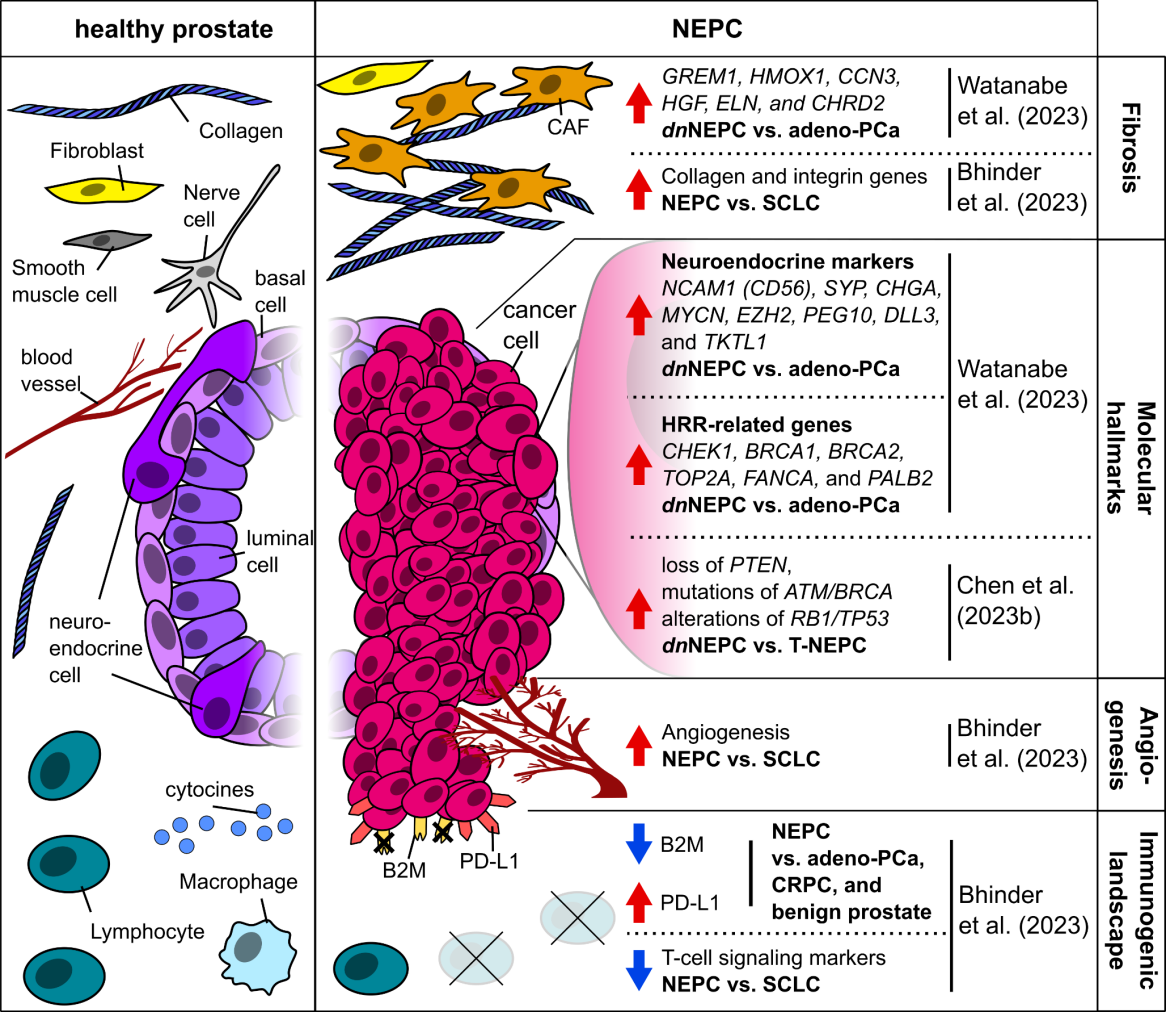
Overview on molecular and TME based alterations in NEPC. Data are summarized from a study (i) employing spatial transcriptomics in a patient with concurrent *de novo* NEPC and adeno-PCa (Watanabe et al. 2023) (ii) analyzing scRNAseq data from multiple prostate cancer cohorts, including patients with 25 pure and 11 mixed NEPC morphology (not specified for *de novo* and T-NEPC) (Bhinder et al. 2023) and (iii) conducting a meta-analysis on gene mutations and copy number alterations in NEPC (specified for *de novo* and T-NEPC) (Chen et al. 2023b). The red and blue arrows indicate an up- or downregulation of the respective attributes in NEPC compared to another indicated cancer. NEPC = neuroendocrine prostate cancer (not specified whether *de novo* and T-NEPC); *dn*NEPC = *de novo* neuroendocrine prostate cancer T-NEPC = treatment-emergent neuroendocrine prostate cancer; adeno-PCa = adenocarcinoma of the prostate; SCLC = small cell lung cancer, HRR = homologous recombinant repair; CRPC = castration-resistant prostate cancer, CAF = cancer-associated fibroblast

MYCN has been also shown to interact with the polycomb recessive complex 2 (PRC2) proteins EZH2 and SUZ12, as well as to suppress AR signaling and increase expression of epithelial-mesenchymal transition genes (Dardenne et al. 2016). Further studies affirmed EZH2 to be an important epigenetic driver of NEPC differentiation in organoids (Puca et al. 2018), that is highly expressed in *de novo* NEPC patient tissue (Fig. 1) (Watanabe et al. 2023). A recent study underlined the importance of epigenetic regulation, such as chromatin remodeling, in the progression of NEPC (Zhang et al. 2024). By developing an algorithm for the risk assessment of NEPC (NEPAL), the authors were able not only to highly precisely identify NEPCs by their gene expression signature, but also to differentiate those into eight NEPC sub-clusters, employing eleven scRNAseq datasets. However, their data also suggest, that of the previously described gene mutations in NEPC, only *TP53* seems to be highly mutated in the predicted high-risk group, while NEPC risk correlated with the expression of DNA methyltransferases (i.e., *DNMT3A*, *DNMT3B*, *DNMT1*) and some members of the PRC2 (e.g. *EZH2* and *RBBP4)* (Zhang et al. 2024). Spatial expression analysis by Watanabe and colleagues furthermore revealed higher expression of the HRR-related genes *CHEK1*, *BRCA1*, *BRCA2*, *TOP2A*, *FANCA*, and *PALB2* in NEPC compared to adeno-PCa (Fig. 1) (Watanabe et al. 2023).

A recent systematic review and meta-analysis further underscored the molecular differences between *de novo* NEPC and T-NEPC (Chen et al. 2023b). *De novo* NEPC was associated with more frequent *PTEN* loss and mutations of *ATM/BRCA*, and more frequent concurrent alterations of *RB1/TP53* compared to T-NEPC (Fig. 1) (Chen et al. 2023b). These findings emphasize the need for a deeper understanding of the molecular distinctions between *de novo* and T-NEPC, as this knowledge could lead to the development of tailored therapeutic approaches.

### The tumor-microenvironment of NEPC

The prostate glandular epithelium is embedded within the stroma, which is composed of extracellular matrix (ECM) proteins, nerves, vascular vessels, fibroblasts, smooth muscle cells and several immune cell types (Fig. 1) (Levesque and Nelson 2018). Furthermore, many stromal-epithelial interactions take place, with stromal cells mediating epithelial development and growth (reviewed by Cunha 2008). Conversely, epithelial cells also influence the differentiation of mesenchymal stroma cells into smooth muscle cells (reviewed by Cunha 2008).

Since *de novo* NEPC is very rare compared to T-NEPC, research of the tumor microenvironment (TME) is mostly focusing on the latter when it comes to NEPC. Generally, recent studies suggest that the progression and the metastatic potential of PCa is strongly influenced by its TME (Andersen et al. 2016; Mo et al. 2018; Brady and Nelson 2023). The TME of PCa harbors several cell types with an immunosuppressive nature (Stultz and Fong 2021). However, early attempts to target the TME with anti-angiogenic agents or immunotherapy have largely failed to meet their primary endpoints in clinical trials (Haidl et al. 2017; Heidegger et al. 2021, 2022), underscoring the need for better characterization of the TME and its interactions with tumor cells.

One major obstacle to the success of immunotherapy is irreversible CD8 + T-cell dysfunction or depletion in the TME (Raskov et al. 2021). A retrospective analysis of RNA and whole exome sequencing data from multiple prostate cohorts revealed that T-cell depletion is more prominent in NEPC than in adenocarcinomas and therefore correlates with aggressiveness of this cancer (Fig. 1) (Bhinder et al. 2023). Interestingly, their data also revealed the existence of a small sub-population of patients with inflamed NEPC, which might be targetable by therapy employing immune checkpoint blockade (ICB) (Bhinder et al. 2023). Moreover, NEPC did show the highest expression of programmed cell death ligand-1 (PDL1), especially in metastatic tumors, compared to other PCa subtypes (Bhinder et al. 2023). Additionally, immune evasion by NEPC was supposed to be enhanced by its significant downregulation of *B2M* relative to other cancers (Bhinder et al. 2023) (Fig. 1), since beta2-microglobulin (B2M), an important component of the MHC-I complex, was previously shown for other cancers to negatively correlate with immune evasion (Wang et al. 2021a).

A previous review summarized multiple studies on how cancer-associated fibroblasts (CAFs), which constitute a heterogeneous group of fibroblasts (Levesque and Nelson 2018; Gao et al. 2024), contribute to an immunosuppressive TME in various cancers by several mechanisms, including hindering effects on T-cell immunity (Koppensteiner et al. 2022). While healthy fibroblasts express many components of the ECM such as collagens, and form lose connections within the stroma (Bonollo et al. 2020), they usually remain in a resting state and promote homeostasis of the prostate (ChallaSivaKanaka et al. 2022). Koppensteiner et al. (2022) summarized that CAFs build up a high-density ECM, constituting a mechanical barrier that hampers the infiltration of a tumor by T-cells. Additionally, CAFs were reported to directly impede CD4 + and CD8 + T-cell proliferation, while also interfering with CD8 + T-cell priming by inhibiting dendritic cell differentiation (Koppensteiner et al. 2022). Altogether, these reports and the observations of the generally immune-cold (Bhinder et al. 2023) and highly fibrotic (Watanabe et al. 2023) NEPC TME, give rise for the need of future research on NEPC.

NEPC also differed from SCLC by higher angiogenesis (Fig. 1) and hedgehog signaling (Bhinder et al. 2023) which are associated with tumor growth and metastasizing (Russo et al. 2012) and tumor progression and poor prognosis (Jing et al. 2023), respectively. Previous studies showed that angiogenesis, as well as tumor growth and progression are strongly influenced by CAFs and their cytokine expression (Bedeschi et al. 2023). On the other hand, based on their gene expression profile CAFs can be subdivided into six subgroups with distinct functions (Luo et al. 2022). Consequently, being the most common cell type within the TME (Koppensteiner et al. 2022), CAFs are composed of the three major subtypes myofibroblasts, inflammatory CAFs, and adipogenic CAFs, as well as endothelial-to-mesenchymal transition CAFs, peripheral nerve-like CAFs, and antigen-presenting CAFs (Luo et al. 2022). In T-NEPC, upregulation of the CHRM4/AKT/MYCN has been linked to an increased levels of interferon alpha 17 (IFNA17) within the TME (Wen et al. 2023). In concordance, the authors observed higher IFNA17 protein and serum levels in AR-negative compared to androgen-dependent prostate cancer cell lines. Furthermore, they described that M2-like macrophages are potential drivers of an immunosuppressive TME, promoting NEPC differentiation with increased PD-L1 expression through IFNA17 and CHRM4 (Wen et al. 2023). In combination with the generally high PD-L1 expression levels in NEPC (Fig. 1) (Bhinder et al. 2023) it is possible that IFNA17 and M2-like macrophages also play a role in the progression of *de novo* NEPC.

About 30% of the ECM are collagens, whereof the fibrillar subtype constitutes 90% (Song et al. 2022). Higher rates of collagen expression (Zhang et al. 2023) as well as fibrotic stiffening of the ECM are observed in many cancers and correlate with their aggressiveness (Piersma et al. 2020). Consequently, specific collagens have already been suggested as biomarkers for a variety of cancers (Song et al. 2022), including the peptides of several collagens (such as collagen alpha-2 type I), which were proposed as potential biomarkers in urine samples from PCa patients (Frantzi et al. 2019, 2023). Our recent study confirmed the correlation between higher collagen expression within the TME with a clinical significance of PCa (Heidegger et al. 2024). Although the available data for *de novo* NEPCs are very scarce, Watanabe and colleagues observed different expression patterns in the TMEs of co-existing *de novo* NEPC and adenocarcinoma in a 78 years old man (Watanabe et al. 2023). Among others, *CHRDL2*, *CCM3*, *GREM1*, *HGF*, and *ELN* were higher, while *RBFOX3*, *PAGE4*, *IGF1* and *SERTM2* were lower expressed in the NEPC compared to the adenocarcinoma TME (Watanabe et al. 2023). Therefore, the TME of NEPC showed high marker expression for fibrosis (Fig. 1) (Watanabe et al. 2023), which generally cause proliferation of CAFs (Ding et al. 2018; Ren et al. 2019; Watanabe et al. 2023). While higher levels of fibrosis markers observed in the TME of NEPC (Watanabe et al. 2023) could also be a possible explanation for NEPCs aggressiveness, further studies of this nature are needed in the future. Not only to gather a representative number of samples, but especially to pinpoint specific attributes of the NEPC TME and to ascertain the differences between this and the TME of adeno-PCa. Since tumor metastasis is heavily influenced by interactions with the TME cellular and extracellular components (Wang et al. 2023), the precise identification and specification of those (such as CAF subgroups and immune cells) will further elucidate which factors drive the aggressive nature of NEPC.

### Conclusion and future perspectives

Even though recent studies focused on NEPC, this cancer - especially when arising *de novo* - still remains poorly understood. However, the advent of innovative technologies such as single cell RNA sequencing or spatial transcriptomics offer unparalleled opportunities to deepen our understanding for biological mechanisms in human disease, in particular in cancer. However, until now only few studies covering NEPC (Bhinder et al. 2023; Watanabe et al. 2023; Chen et al. 2023b) exist, which is why there remains a substantial gap in our knowledge regarding the highly aggressive NEPC. This is mostly due to *de novo* NEPC being a very rare and T-NEPC only emerging in at most about 30% upon treatment of adeno-PCa (Le et al. 2023; Liu et al. 2022). In general, NEPC is characterized by rapid metastasis and currently has very limited therapeutic options.

Revision of the recent literature further highlighted that there is a need to thoroughly indicate NEPC subtypes (e.g. *de novo* or treatment-emergent). This is of high importance, not only because several phenotypic subtypes with different molecular hallmarks have already been described for NEPC (Cejas et al. 2021; Chen et al. 2023a; Wang et al. 2022). In addition, while one study reported that NEPCs are generally immune-cold with only a small number being inflamed and infiltrated by T-cells (Bhinder et al. 2023), it remains elusive how exactly molecular hallmarks of NEPC cells are intertwined with the cellular compartments of the TME. Thus, a thorough investigation of NEPC and its TME is crucial, as understanding the unique interactions and cellular changes within this environment could pave the way for developing more effective treatments. They include but are not limited to a better understanding of the specific molecular pathways involved in T-cell depletion, PD-1/PD-L1 expression, CAF-mediated immune suppression and novel insights in the highly heterogeneous macrophage biology within cancers.

Innovative high resolution and high throughput technologies are highly beneficial for the examination of rare malignancies like NEPC. Their application in tumor/TME research holds the promise of uncovering critical molecular and cellular dynamics that drive its aggressive behavior and resistance to existing therapies. This would lead to the identification of novel biomarkers, therapeutic targets and treatment strategies, ultimately improving prognosis and survival rates for patients affected by this challenging subtype of PCa.

## Data Availability

No datasets were generated or analysed during the current study.
